# Supplementary material for: Controlled beat-wave Brillouin scattering in the ionosphere
Source: Nat Commun. 2021 Oct 27;12:6209. doi: 10.1038/s41467-021-26305-9 (PMC8551208; doi:10.1038/s41467-021-26305-9)
Supplement: Supplementary file 1 — Supplementary Information [file 41467_2021_26305_MOESM1_ESM.pdf]

Supplementary Information

Controlled beat-wave Brillouin scattering in the ionosphere

B. Eliasson[1*], A. Senior[2], M. Rietveld[3,†], A.D.R. Phelps[1], R.A. Cairns[4], K. Ronald[1], D.C. Speirs[1], R.M.G.M. Trines[5], I. McCrea[5], R. Bamford[5], J. T. Mendonça[6], and R. Bingham[1,5]

[1]Department of Physics, SUPA, University of Strathclyde, Glasgow, G4 0NG, United Kingdom
[2]Independent Researcher, Lancaster, United Kingdom
[3]EISCAT, Ramfjordmoen, N-9027 Ramfjordbotn, Norway
[4]School of Mathematics and Statistics, University of St Andrews, St Andrews, KY16 9SS, United Kingdom
[5]STFC Rutherford Appleton Laboratory, Harwell, Oxford, Didcot, OX11 0QX, United Kingdom
[6]Instituto Superior Técnico, Av. Rovisco Pais, Nº 1, 1049-001 Lisboa, Portugal
†also at Department of Physics and Technology, UiT, The Arctic University of Norway, 9037 Tromsø, Norway
* email: bengt.eliasson@strath.ac.uk

21 September 2021

Ionospheric conditions during the experiment

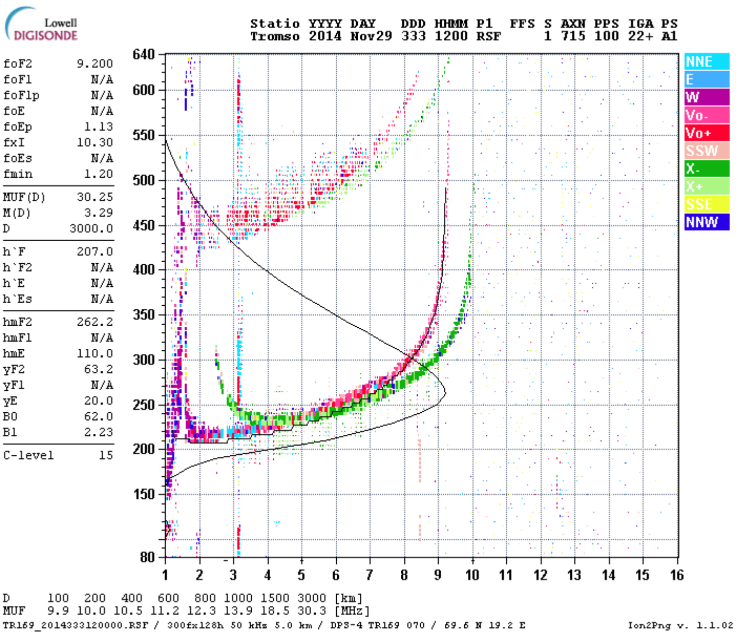

Supplementary Figure 1 | Ionogram from Tromsø on 29 Nov. 2014. The ionogram [1] was recorded at 12:00 UTC (during Run V). The electron plasma frequency is indicated by a solid line, with a maximum value $\mathrm{FoF2} = 9.2$ MHz at the altitude 262.2 km.

A typical ionogram [1] taken during the experiment (run V) at 12:00 UTC is shown in Supplementary Figure 1, where it is seen that the critical frequency of the F2 layer $\mathrm{FoF2} = 9.2$ MHz. Vertically injected O mode waves in the frequency range 6 – 8 MHz would be reflected at altitudes of 215 – 230 km. The magnetic field strength B_0 in Tromsø is 53.10 µT on ground and 46.95 µT at 300 km [2]. At an altitude of 220 km, the magnetic field is estimated to be $B_0 \approx 48.59\ \mu\mathrm{T}$ and the corresponding electron cyclotron frequency $f_{ce} \approx 1.36$ MHz. At Tromsø, the angle of the magnetic field to the vertical is $\alpha \approx 11.8°$.

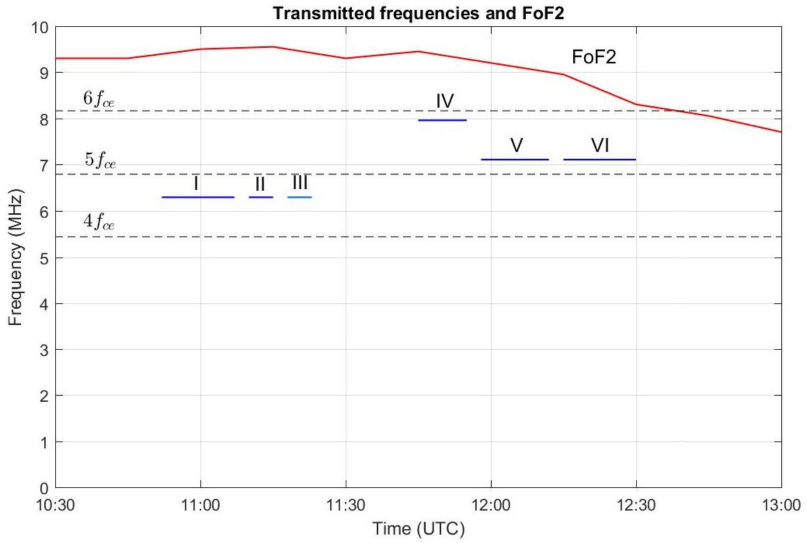

Supplementary Figure 2 | Transmitted frequencies and ionospheric critical frequency. The time variation of the critical frequency of the F2 layer $\mathrm{FoF2}$ (red lines), the transmitted frequencies f_0 in Runs I – VI (blue horizontal lines), and the 4th – 6th harmonics of the electron cyclotron frequency f_{ce} (horizontal dashed lines).

Using data from available ionograms [1], Supplementary Figure 2 shows the time-variation of $\mathrm{FoF2}$ compared to the times and the frequencies of Runs I – VI. It is noted that Run IV (7.953 MHz) is slightly below the 6th electron cyclotron harmonic, while Runs V and VI are somewhat above the 5th cyclotron harmonic. It is seen that $\mathrm{FoF2}$ was somewhat above 9 MHz during most of the experiments but was dropping at the end.

Transmitter and receiver setup

To avoid saturation by the direct signal from the transmitter (if reception of the probe was to be carried out at Ramfjordmoen using the HF receiver), the remote SEE receiver [3,4] installed at Kroken was instead used to record the signal. As seen in Figure 1 of the main article, Kroken is located near Tromsø, about 13 km north-northwest (NNW) of Ramfjordmoen separated by a mountainous region. Since the down-coming probe wave and SEE sidebands were not very weak, the modest antenna used by the SEE receiver was deemed adequate. The experiment used continuously-radiated pump and probe waves, with the frequency of the probe being stepped between a frequency far enough (100 Hz) from the pump to prevent resonant Brillouin interaction, and several different frequencies close to the pump where resonant interaction can occur. The received signals were then analysed and compared between these different offset frequencies from the pump.

Beam patterns for EISCAT array 1 are shown in Supplementary Figure 3 for frequency 7.1 MHz and for O and X polarization. The vertically directed heater beam has a typical width of about 6.5°-7.0°; hence, most of the injected power is injected at incidence angles smaller than the Spitze angle [5], $|\theta| < \theta_{\mathrm{c}}$, where $\theta_{\mathrm{c}} = \arcsin\left[\sqrt{Y/(1+Y)}\sin\alpha\right]$ (with $Y = f_{\mathrm{ce}}/f_0$) is between $4.5°$ and $5°$ for a transmitted frequency $f_0 = 6 – 8$ MHz. At certain injection angles, the O mode wave is linearly converted to plasma waves that remain trapped in the plasma (cf. Section 4 of Ref. 5). In a narrow cone around the Spitze angle $\theta = \theta_{\mathrm{c}}$, the O mode is converted to an upward propagating Z mode wave that is reflected at a higher altitude and then propagates down toward its electrostatic resonance during which its wavelength decreases and it becomes increasingly electrostatically polarized as it is transformed to an electrostatic Langmuir wave. The electrostatic resonance is

located about 65 meters below the $X = 1$ level in Figure 5b of the main article, where the dielectric tensor component $\epsilon_{zz} = 0$, with $\epsilon_{zz} = 1 - X(1 - Y^2 \cos^2 \alpha)/(1 - Y^2)$, giving $X \approx 0.9990$. The direct coupling between the upward propagating O mode and downward propagating Langmuir wave is very weak since the two waves have vastly different wavelengths and different polarizations; the O mode has transverse polarization with the electric field perpendicular to the wave vector while the Langmuir wave is longitudinal with the electric field parallel to the wave vector [6]. At the negative Spitze angle $\theta = -\theta_c$, the O mode reaches a turning point slightly above the $X = 1$ level after which it propagates down to the electrostatic resonance where it is gradually converted to a Langmuir wave. A larger amount of O mode power will be converted to upper hybrid waves on striations at the upper hybrid layer and to Langmuir turbulence at the critical layer. At $|\theta| > \theta_c$ as well as at directions off the magnetic meridian plane, the O mode wave is reflected at an altitude below the critical layer, and when reflected below the upper hybrid layer, the conversion to electrostatic waves is diminished.

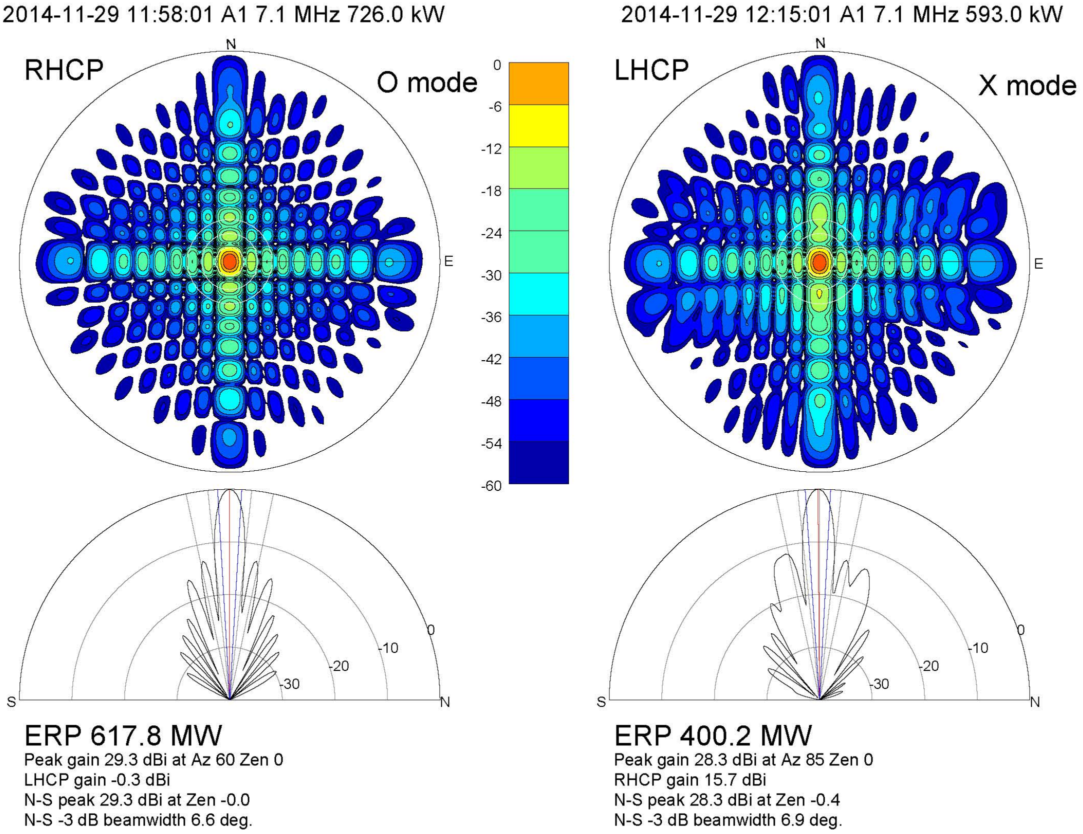

Supplementary Figure 3 | Beam patterns for EISCAT array 1. Shown beam patterns and ERP are for frequency 7.1 MHz and for O and X mode polarization (Runs V and VI).

The antenna beam patterns plotted in Supplementary Figure 3 and the estimated ERPs in Table 1 of the main article were obtained using the 'heatererp' software (https://gitlab.com/andrewsenior/heatererp) written by A. Senior with contributions by M. T. Rietveld. More details on the ERP calculations are given in Section 6.6 of Ref. 7. Beam patterns for several other cases can be found at the University of Strathclyde repository (https://doi.org/10.15129/d4ca75d2-a462-4940-9614-b1b3fe5a8e2e).

Kinetic model of ion-acoustic (IA) waves.

For ion-acoustic (IA) waves propagating at small angles to the magnetic field lines, the magnetic field effects may be neglected (see below). The frequency then approximately follows the dispersion relation in fluid theory for un-magnetised plasma, $2\pi f_{\mathrm{IA}} = \omega_{\mathrm{IA}} \approx C_{\mathrm{s}} k_{\mathrm{IA}}$, where $C_{\mathrm{s}} = \sqrt{k_{\mathrm{B}}(T_{\mathrm{e}} + 3T_{\mathrm{i}})/m_{\mathrm{i}}}$ and we consider oxygen ions (O^{+}) with mass $m_{\mathrm{i}} = 16u$. However, the IA waves suffer collision-less damping (Landau damping) through resonant interaction between the wave and the particles. In kinetic theory, the dispersion relation reads

$$1 + \chi_{\mathrm{i}} + \chi_{\mathrm{e}} = 0, \tag{S1}$$

where the respective ion and electron susceptibilities are obtained from kinetic theory as

$$\chi_{\mathrm{i}} = \frac{\omega_{\mathrm{pi}}^2}{v_{\mathrm{Ti}}^2 k_{\mathrm{IA}}^2}\big(1 + \xi_{\mathrm{i}} \mathrm{Z}(\xi_{\mathrm{i}})\big), \tag{S2}$$

$$\chi_{\mathrm{e}} = \frac{\omega_{\mathrm{pe}}^2}{v_{\mathrm{Te}}^2 k_{\mathrm{IA}}^2}\big(1 + \xi_{\mathrm{e}} \mathrm{Z}(\xi_{\mathrm{e}})\big). \tag{S3}$$

Here,

$$\mathrm{Z}(\xi) = e^{-\xi^2}\left(i\sqrt{\pi} - 2\int_0^{\xi} e^{t^2}\, dt\right) \tag{S4}$$

is the plasma dispersion function [8], and we denoted $\xi_{\mathrm{i}} = \omega_{\mathrm{IA}}/\big(\sqrt{2} v_{\mathrm{Ti}} k_{\mathrm{IA}}\big)$ and $\xi_{\mathrm{e}} = \omega_{\mathrm{IA}}/\big(\sqrt{2} v_{\mathrm{Te}} k_{\mathrm{IA}}\big)$ where $v_{\mathrm{Ti}} = \sqrt{k_{\mathrm{B}} T_{\mathrm{i}}/m_{\mathrm{i}}}$ and $v_{\mathrm{Te}} = \sqrt{k_{\mathrm{B}} T_{\mathrm{e}}/m_{\mathrm{e}}}$ are the ion and electron thermal speeds, and $\omega_{\mathrm{pi}} = \sqrt{n_0 e^2/\epsilon_0 m_{\mathrm{i}}}$ and $\omega_{\mathrm{pe}} = \sqrt{n_0 e^2/\epsilon_0 m_{\mathrm{e}}}$ are the ion and electron angular plasma frequencies, respectively. The IA wavenumber k_{IA} is assumed to be real, while the wave frequency is assumed complex valued, $\omega_{\mathrm{IA}} = \omega_{\mathrm{R}} + i\omega_{\mathrm{I}}$, with $i = \sqrt{-1}$ being the imaginary unit.

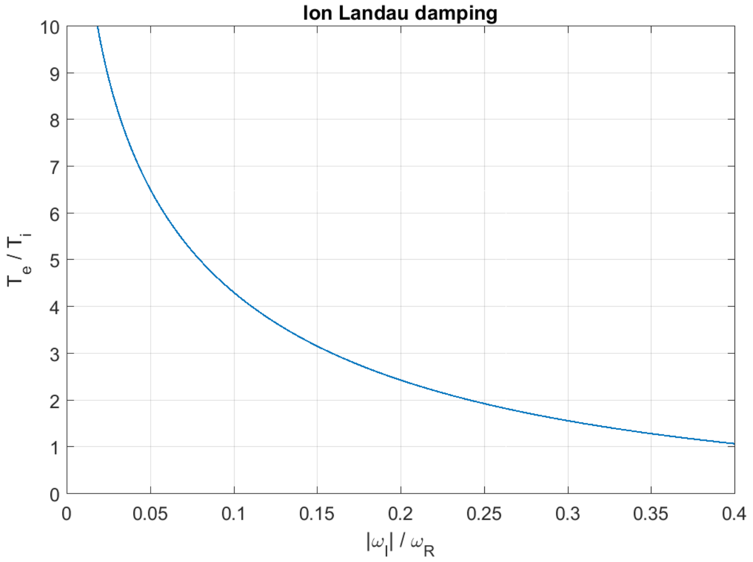

Supplementary Figure 4 | Ion Landau damping. $|\omega_{\mathrm{I}}|/\omega_{\mathrm{R}}$ as a function of the electron-to-ion temperature ratio $T_{\mathrm{e}}/T_{\mathrm{i}}$ for IA waves in the quasi-neutrality limit.

In the long wavelength limit, the IA waves are dispersion-free, and the ratio $|\omega_{\mathrm{I}}|/\omega_{\mathrm{R}}$ depends only on $T_{\mathrm{e}}/T_{\mathrm{i}}$, as shown in Supplementary Figure 4. The IA waves suffer strong Landau damping when the ion and electron temperatures are comparable. For X mode heating with $T_{\mathrm{e}}/T_{\mathrm{i}} = 1.5$ gives $|\omega_{\mathrm{I}}| \approx 0.31\omega_{\mathrm{R}}$, while for O mode heating the peak electron temperature $T_{\mathrm{e}} = 3500$ K near the reflection height gives $T_{\mathrm{e}}/T_{\mathrm{i}} = 3.5$ and a local decrease of the ion Landau damping to $|\omega_{\mathrm{I}}| \approx 0.13\omega_{\mathrm{R}}$.

At IA frequencies comparable to or lower than the ion cyclotron frequency ($f_{\mathrm{ci}} \approx 45$ Hz at EISCAT), the ion motion is influenced by the magnetic field. Fluid theory gives the IA dispersion relation (omitting Landau damping) [9]

$$\omega_{\mathrm{IA}}^4 - \left(\omega_{\mathrm{ci}}^2 + C_s^2 k_{\mathrm{IA}}^2\right)\omega_{\mathrm{IA}}^2 + \omega_{\mathrm{ci}}^2 C_s^2 k_{\mathrm{IA}}^2 \cos^2\theta = 0, \quad \text{(S5)}$$

where θ is the angle between the IA wave vector and the magnetic field lines, $\omega_{\mathrm{ci}} = eB_0/m_{\mathrm{i}}$ is the ion cyclotron angular frequency, B_0 is the magnetic field strength, and e is the unit charge. The dispersion relation shows that the IA wave has a resonance ($k_{\mathrm{IA}} \to \infty$) at $\omega_{\mathrm{IA}} = \omega_{\mathrm{ci}}\cos\theta$, a cutoff ($k_{\mathrm{IA}} \to 0$) at $\omega_{\mathrm{IA}} = \omega_{\mathrm{ci}}$, and a forbidden frequency band between the resonance and cutoff. The branch at $\omega_{\mathrm{IA}} < \omega_{\mathrm{ci}}$ is the magnetized IA wave, while the higher frequency branch $\omega_{\mathrm{IA}} > \omega_{\mathrm{ci}}$ is the electrostatic ion cyclotron wave. The scattering of the EM wave off these wave modes and off ion Bernstein waves are referred to as magnetized stimulated Bernstein scattering (MSBS), and occurs primarily where the EM wave propagates at oblique angles to the magnetic field and the transmitted frequency is not too close to electron cyclotron harmonics so that magnetic field aligned striations can be formed [4,9-11]. In stimulated ion Bernstein scattering (SIBS), which appears when the O mode frequency is tuned near an electron cyclotron harmonic [12,13], the EM wave is converted into a high-frequency upper hybrid wave on a low-frequency ion Bernstein wave propagating at large angles to the magnetic field, in a process similar to the parametric decay instability (PDI) discussed below. However, for IA waves propagating at small angles to the magnetic field, the effect of the magnetic field on the IA wave may be neglected except very close to the cyclotron resonance, and the dispersion relation (S5) simplifies to $\omega_{\mathrm{IA}}^2 \approx C_s^2 k_{\mathrm{IA}}^2$. With this assumption, the magnetic field effects on the IA wave propagation have for simplicity been neglected in the kinetic description and numerical simulations of the IA waves.

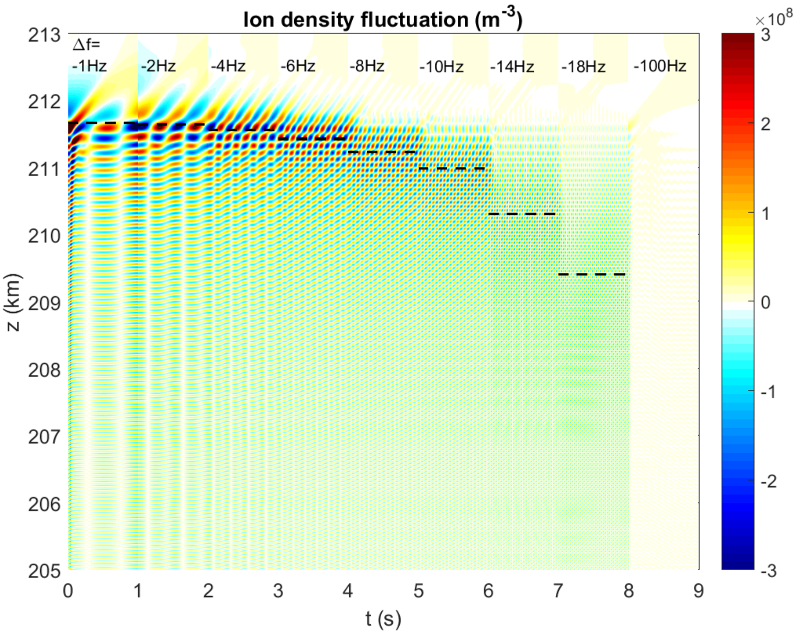

Supplementary Figure 5 | Spatiotemporal evolution of ion density. Ion density fluctuation n_{i1} as a function of altitude and time for combined pump and probe X mode. Resonant heights where $-\Delta f = f_{\mathrm{IA}}$ are indicated by horizontal dashed lines, with the value of Δf (Table 2 of the main article) indicated for each 1-second time slot.

The ion acoustic frequency is related to the IA wavelength through $f_{\mathrm{IA}} \approx C_s/\lambda_{\mathrm{IA}}$, and at resonant interaction the IA wavelength is approximately half the wavelength of the electromagnetic (EM) wave, $\lambda_{\mathrm{IA}} = \lambda_{\mathrm{EM}}/2$. The resonant interaction between the beat-wave and the IA wave occurs at an altitude where $-\Delta f = f_{\mathrm{IA}}$, where f_{IA} [Figure 4(e) of the main article] increases continuously from zero at the critical layers of the O and X mode waves to about 65 Hz at low altitudes approaching free space conditions where $\lambda_{\mathrm{EM}} \approx c/f_0 = 47.6$ m and $C_s \approx 1.53 \times 10^3$ m/s. The simulated spatiotemporal evolution of the ion density fluctuations n_{i1} driven by the X mode beat-wave is shown in Supplementary Figure 5. For visibility the time-averages over 1s have been subtracted in

each of the one-second time-slots. The ion fluctuations are strongest at the resonant heights where $-\Delta f = f_{\rm IA}$, indicated by horizontal dashed lines in Supplementary Figure 5. The resonant layer progressively moves to lower altitudes for larger Δf, and the ion fluctuations become weaker. For small values of Δf, the interaction takes place in a thin layer very close to the reflection point $z = 211.68\ \rm km$, where the spatial profile of the electromagnetic wave is best described by Airy functions [6,14] and not as a sinusoidal wave. Therefore the wave vector matching condition (1b) breaks down and the character of the interaction may change from a three-wave to a four-wave interaction where the electromagnetic wave is modulated in amplitude by the slowly time-varying ion density fluctuations [15]. For $t = 8$ - 9 s, the beat frequency $\Delta f = -100\ \rm Hz$ is of larger magnitude than the maximum resonant IA frequency $f_{\rm IA} \approx 65\ \rm Hz$ [cf. Figure 4(e) of the main article], leading to non-resonant interaction. In Figure 2 of the main article, Runs II and III have clearly visible upshifted sidebands at $+100$ Hz for $\Delta f = -100\ \rm Hz$ indicating non-resonant interaction, while this sideband is weaker for other runs. Oxygen (O^+) and nitric oxygen (NO^+) ions have their gyrofrequencies at 46.6Hz and 24.8Hz, respectively, and their harmonics could potentially be involved in the enhancement at +100 Hz. However, such an upshifted sideband is also visible in the simulated spectrogram in Figure 7a of the main article for the pump+probe case, using un-magnetized ions, supporting the experimental observations without ion cyclotron harmonic effects. As mentioned in the main article, repetition of the frequency spectra at multiples of 50 Hz in Figure 2 of the main article is attributed to weak radiated sidebands at 50 Hz and harmonics thereof because of imperfect power supply filtering.

Collisional and anomalous absorption of EM waves

In linear theory [16,17], EM waves can be absorbed due to electron collisions with ions and neutrals. For a relatively weak magnetic field ($Y \ll 1$) in a weakly collisional plasma, the decrement per meter of the EM wave amplitude may to first order be estimated as

$$\kappa = \frac{\nu_{\rm e} X}{2cn}, \tag{S6}$$

where $n = ck/\omega$ is the the refractive index obtained from the Appleton-Hartree dispersion relation [cf. Eq. (5) in the main article], and where the electron collision frequency $\nu_{\rm e} = \nu_{\rm en} + \nu_{\rm ei}$ is the sum of the electron-neutral and electron-ion collision frequencies $\nu_{\rm en}$ and $\nu_{\rm ei}$. For typical ionospheric parameters, electron-neutral collisions dominate in the D and E regions below about 150 km while electron-ion collisions dominate at higher altitudes in the F region [9]. For propagation in the ionosphere, the relative EM wave amplitude may then be estimated as $\sim \exp\left(-\int \kappa\, ds\right)$ where s is the path length. Linear absorption of power due to electron-neutral collisions in the D region (below 90 km) is typically 6-10 dB during daytime conditions when the electron number density is higher at lower altitudes where the neutral gas is denser, but the absorption is much lower during night-time conditions [18]; at higher altitudes in the E and F regions the electrons are weakly collisional and linear absorption less significant. Typically $\nu_{\rm e} < 10^3\ \rm s^{-1}$ in the F2 region giving a very small $\kappa \sim 10^{-6} - 10^{-5}\ \rm m^{-1}$.

However, experiments show that a large amplitude O mode wave can undergo absorption in the F2 region that is much greater than collisional absorption [18-22]. When first discovered, this additional absorption was called anomalous absorption, a term which is now typically understood to mean absorption through parametric instability processes. During a certain time after arrival of the leading edge of the reflected pump signal, the intensity of the reflected signal remains almost constant. This corresponds to the linear growth phase of the instabilities. The duration of the linear phase is of the order 4-5ms at an injected ERP of 12 MW but is significantly shorter 0.1–0.2 ms for a higher ERP of 1.7 GW [23]. After the linear phase, anomalous absorption sets in, and the intensity of the reflected signal abruptly drops by about 10-30 dB, with higher absorption at higher injected power. During the linear phase, the wave electric field of the O mode becomes aligned with the background magnetic

field (i.e. almost in the vertical direction at Tromsø) as the wave reaches the critical layer [6,14], where it excites short wavelength electrostatic Langmuir turbulence via the oscillating two-stream instability (OTSI) and parametric decay instability (PDI) [6] with the electrostatic waves propagating primarily parallel to the magnetic field lines. The PDI is a three-wave process in which the EM wave resonantly drives a high-frequency Langmuir wave and a low-frequency propagating IA wave which oscillates while increasing its amplitude with time. The OTSI was originally studied by Nishikawa [24] who considered a Langmuir wave as the pump wave, and was shown to be a purely growing four-wave instability in which a non-resonant density perturbation increases monotonically with time. Its name originates from an analogy with a two-stream instability between electrons and ions [25]. When the Langmuir wave is the pump, the OTSI is a modulational instability in which the Langmuir pump wave is modulated in amplitude by the slowly time-varying ion density perturbations. It was later adopted by the laser interaction community as a four-wave non-resonant instability with a monotonically growing ion perturbation, but driven by a transverse wave i.e. the laser beam, and also by the ionospheric community for EM waves in the radio frequency range. This makes it distinct from the Langmuir-driven case. The EM wave-driven OTSI drives two counter-propagating electrostatic Langmuir waves and a monotonically growing density perturbation [26,27] typically having a wavelength λ_L much shorter than that of the EM wave. When the counter-propagating Langmuir waves have grown to large amplitude, they can in turn beat and drive monotonically growing ion density fluctuations at half their wavelength, $\lambda_{\mathrm{L}}/2$. The growing ion density fluctuations saturate by nucleation with localized Langmuir wave packets trapped in ion density cavities [28,29] resulting in strong, cavitating Langmuir turbulence [27,30-32]. The turbulence leads to intriguing time and space dynamics of the ionospheric interaction region with overshoot phenomena on different time scales as studied using high resolution incoherent backscatter radar observations with possible explanations for these effects [32,33].

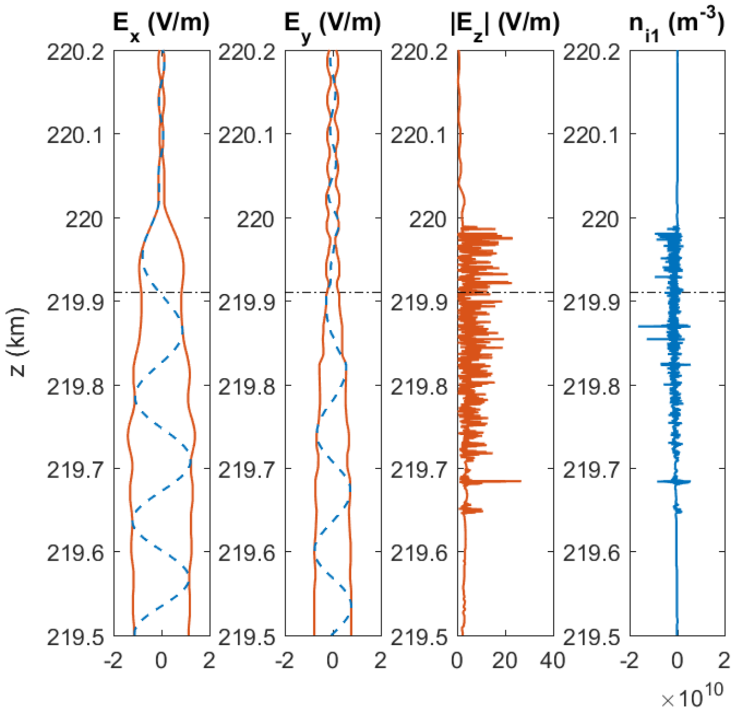

Supplementary Figure 6 | Anomalous absorption of O mode wave. Close-up of the electric field and ion density fluctuations near the critical altitude at time 10s (cf. Fig. 6b in the main article). Red lines indicate the amplitude of the electric field components, while blue dashed lines show the wave-form of the O mode electric field. Horizontal dash-dotted lines indicate the unperturbed critical altitude of the O mode.

The mode conversion of the EM wave to high-frequency electrostatic waves works effectively as an anomalous resistivity [34-37] that absorbs the EM wave. Supplementary Figure 6 shows a close-up of the electric field and ion density fluctuations $n_{\mathrm{i}1}$ near the critical layer of the O mode, exhibiting significant damping of the O mode in the region with ion density fluctuations and large amplitude Langmuir wave oscillations in E_z. For a driving electric field parallel to a group of ion waves having

amplitudes n_k for different wavenumbers k, the collision frequency ν_e in Eq. (S6) may be replaced by an effective collision frequency [36,37]

$$\nu_{\rm eff} = \frac{\omega_0}{2}\sum_k \left(\frac{n_k}{n_{\rm i0}}\right)^2 \frac{{\rm Im}(\epsilon_{\rm L})}{|\epsilon_{\rm L}|^2}, \tag{S7}$$

where ${\rm Im}(\epsilon_{\rm L})$ denotes the imaginary part of the dielectric function for Langmuir waves. In a fluid description, $\epsilon_{\rm L}(\omega_0, k) = 1 + \omega_{\rm pe}^2/\left(3v_{\rm Te}^2 k^2 - \omega_0(\omega_0 + i\nu_{\rm e})\right)$. For a continuous spectrum of ion fluctuations the sum is replaced by an integral. An order-of-magnitude estimate gives $\nu_{eff} \sim 0.1\omega_0(\lambda_{\rm De}k_{\rm L})^{-2}\langle\delta n_{\rm i1}^2\rangle/n_{\rm i0}^2 = 0.01\omega_0 = 4\times10^5\ {\rm s}^{-1}$ for typical values $\lambda_{\rm De}k_{\rm L} = 0.03$ and $\langle\delta n_{\rm i1}^2\rangle/n_{\rm i0}^2 \sim 10^{-4}$ in Supplementary Figure 6, and using the transmitted frequency $\omega_0 = 2\pi\times6.3\times10^6\ {\rm s}^{-1}$. Here $\lambda_{\rm De}$ is the electron Debye length, $k_{\rm L} = \left(\omega_0^2 - \omega_{\rm pe}^2\right)^{1/2}/(\sqrt{3}\,v_{\rm Te})$ is the local wavenumber of the Langmuir wave obtained from the Bohm-Gross dispersion relation, and $\langle\delta n_{\rm i1}^2\rangle = \sum_k n_k^2$ is the mean square of the ion density fluctuations. Hence, the anomalous resistivity due to ion fluctuations can be at least 3 orders of magnitude higher than that due to collisions. As the O mode approaches the critical layer, its group speed decreases and it can spend significant time in the region with small-scale ion density fluctuations, leading to almost complete anomalous absorption within only a few wavelengths of the O mode, as seen in Supplementary Figure 6. On a much longer timescale of the order of a second, there is often a further drop of about 10-15 dB of the observed reflected power [38-40], which is attributed to the development of magnetic field aligned density striations through thermal instabilities [30,41-43] at the upper hybrid layer, a few km below the critical layer. The striations are typically cylindrical in shape with a few meters radius across the magnetic field but extending several tens of km along the magnetic field lines. An O mode wave injected vertically at Tromsø has most of its wave electric field directed across the striations as it reaches the upper hybrid layer, and is then anomalously absorbed through the conversion to high-frequency upper hybrid waves trapped in the striations [20-22]. The large amplitude trapped upper hybrid waves interact with low-frequency lower hybrid waves to generate upper hybrid and lower hybrid turbulence with the electrostatic waves propagating primarily across the magnetic field lines. Through the anomalous absorption at the upper hybrid layer, a large part of the O mode waves are prevented from reaching the critical layer at higher altitude. However, the formation of striations is suppressed when the transmitted frequency is close to electron gyroharmonics [44,45] due to linear dispersion effects that restrict the existence of upper hybrid waves involved in the formation of the striations [46-48], in which case the O mode can reach and excite Langmuir turbulence at the critical layer. The electrostatic Langmuir and upper hybrid waves dissipate their energy via collisions and wave-particle interactions to heat the electrons and to lesser extent the ions.

Stimulated electromagnetic emissions (SEE) in ionospheric heating experiments

While the SBS processes discussed in this paper give rise to sidebands tens of Hz away from the pump frequency, there are many other types of emissions that have been observed during ionospheric heating experiments with sidebands up to several tens of kHz below or above the pump frequency. The PDI/OTSI and resulting Langmuir turbulence near the critical layer gives rise to the narrow downshifted continuum a few kHz below the pump frequency [49,50] (also seen in the widening of the simulated spectrum in green/yellow in Figure 7b in the main article), while the downshifted maximum and broad upshifted maximum originate from the upper hybrid layer once magnetic field aligned striations have developed [51]. The downshifted maximum is about 10 kHz (the lower hybrid frequency) below the pump frequency and is attributed to upper hybrid and lower hybrid turbulence. The broad upshifted maximum is several tens of kHz above the pump frequency and is generated at the upper hybrid layer due to the coupling to electron Bernstein waves at frequencies between electron gyroharmonics. These and several other types of SEE are covered extensively in review articles [18,23,52-54] and are used as a tool to study ionospheric parameters

and on-demand magnetized plasma turbulence. For example, the downshifted peak and broad upshifted maximum are diminished for pump frequencies very close to electron gyroharmonics due to the suppression of upper hybrid waves near the gyroharmonics [46-48], which gives information about the local magnetic field strength at the upper hybrid layer and indicates that magnetic field aligned striations are suppressed at these frequencies. There are also rare observations of emissions near half the pump frequency $f_0/2$ (as well as at $2f_0$) [55,56] which are attributed to absolute stimulated Raman and/or two-plasmon decay instabilities taking place near the altitude of the quarter-critical density where $\omega_{\mathrm{pe}} = f_0/2$. The Raman instability involves an EM pump wave decaying into a Langmuir wave and an EM sideband while in the two-plasmon decay instability the EM wave decays into two Langmuir waves.

Spectrograms from experimental cycles

The available spectrograms from experimental cycles 29 November 2014 are shown in Supplementary Figures 7-9.

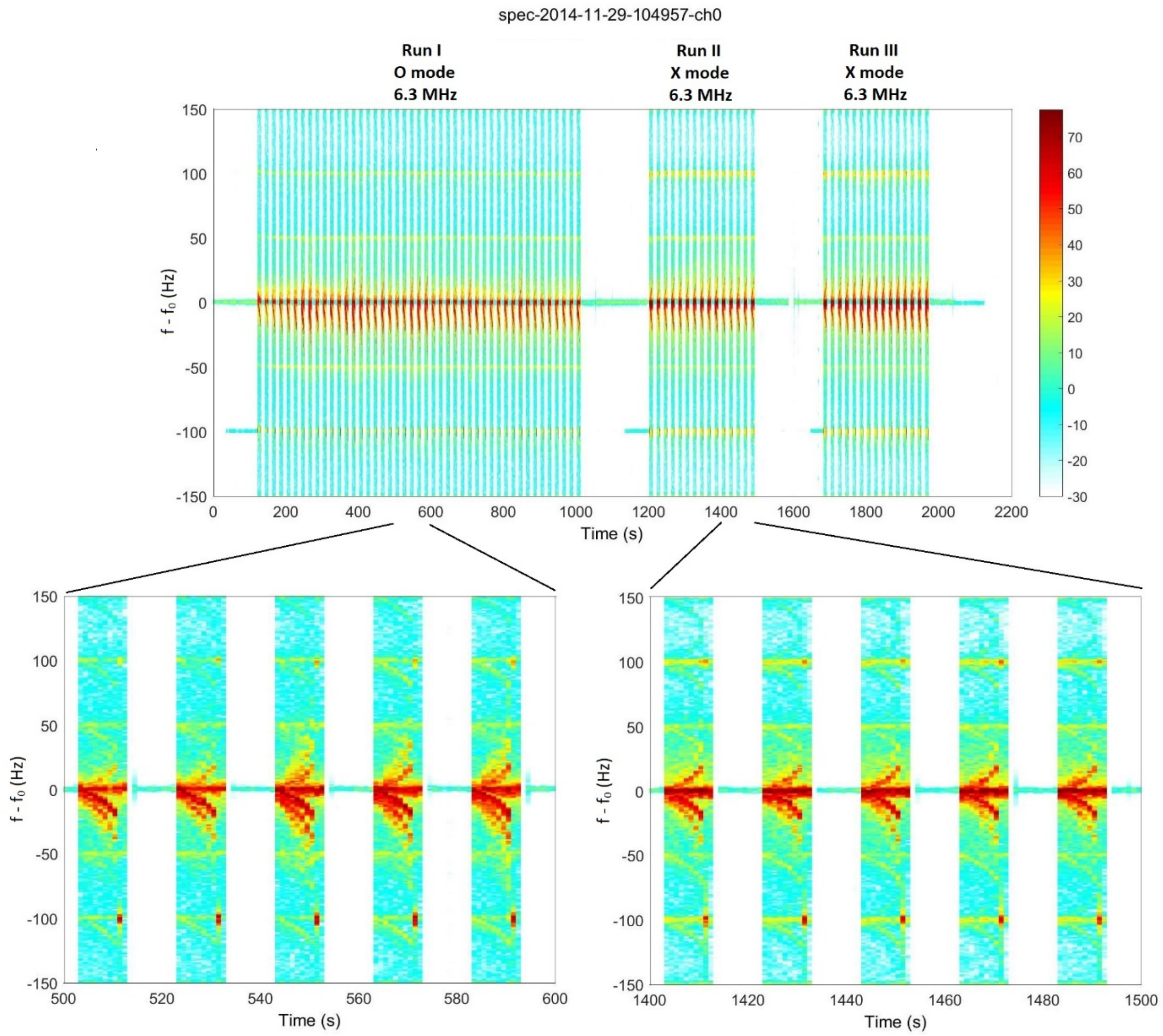

Supplementary Figure 7: Spectrograms of Run I (O mode), Run II (X mode), and Run III (X mode), $f_0 = 6.3$ MHz. Full spectrogram (top) and close-ups of experimental cycles in the time ranges 500-600 s and 1400-1500 s (bottom).

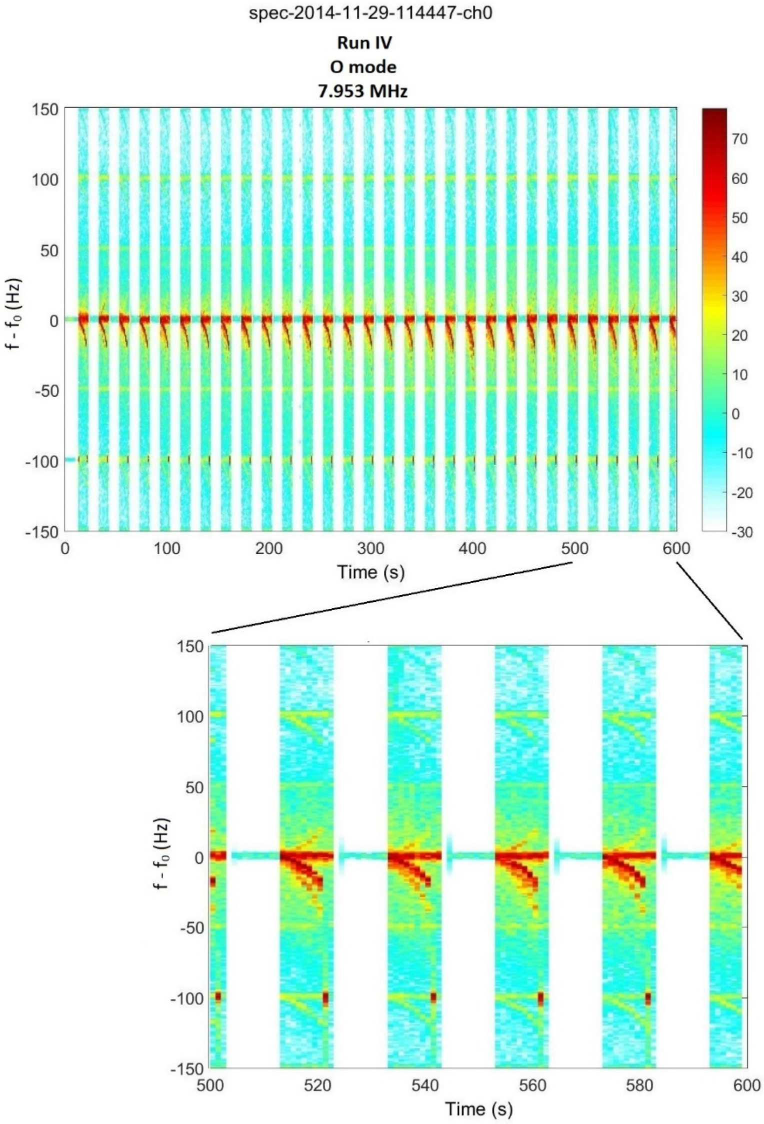

Supplementary Figure 8 | Spectrogram of Run IV (O mode), 7.953 MHz. Full spectrogram (top) and close-up of experimental cycles in the time range 500-600 s (bottom).

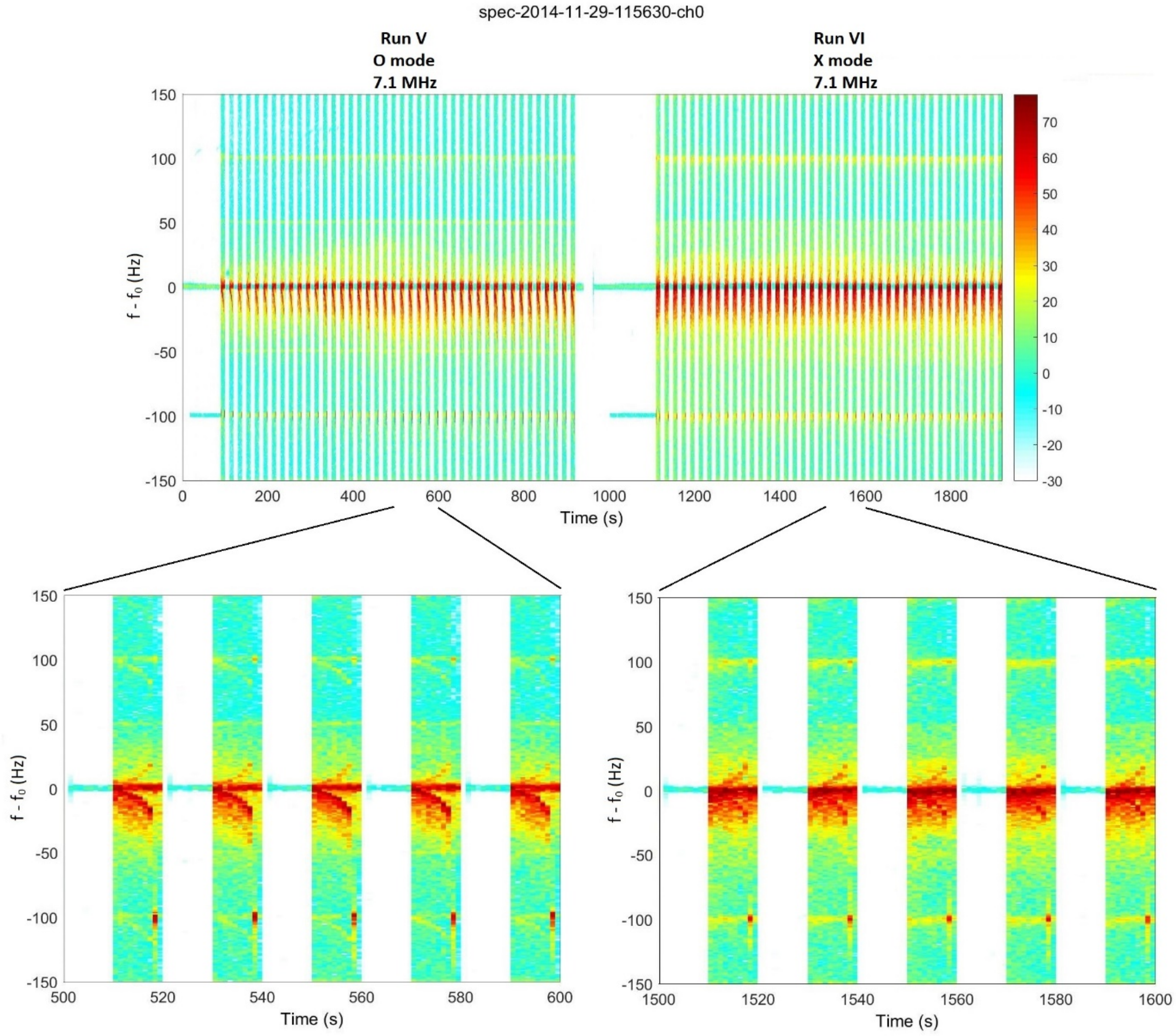

Supplementary Figure 9 | Spectrograms of Run V (O mode) and Run VI (X mode), $f_0 = 7.1$ MHz. Full spectrogram (top) and close-ups of experimental cycles in the time ranges 500-600 s and 1500-1600 s (bottom).

Additional information

Extended data is available for this paper at the University of Strathclyde repository (https://doi.org/10.15129/d4ca75d2-a462-4940-9614-b1b3fe5a8e2e).
Correspondence and requests for materials should be addressed to B.E.

References

[1] Tromsø Geophysical Observatory, Archive ionograms Tromsø 2003 -> today (http://geo.phys.uit.no/ionodata/ionosonde.html). University of Tromsø, Tromsø, Norway.

[2] EISCAT, Technical data of EISCAT locations (https://www.eiscat.se/scientist/document/location-of-the-eiscat-facilities/). EISCAT Scientific Association, Kiruna, Sweden.

[3] Fu, H. Y. et al. Stimulated Brillouin scattering during electron gyro-harmonic heating at EISCAT, *Ann. Geophys.* **33**, 983–990 (2015). https://doi.org/10.5194/angeo-33-983-2015

[4] Fu, H. Y., et al. Electron temperature inversion by stimulated Brillouin scattering during electron gyroharmonic heating at EISCAT. *Geophys. Res. Lett.* **47**, e2020GL089747 (2020). https://doi.org/10.1029/2020GL089747

[5] Mjølhus, E. On linear conversion in a magnetized plasma, *Radio Sci.* **25**(6), 1321–1339 (1990). https://doi.org/10.1029/RS025i006p01321

[6] Mjølhus, E., Helmersen E. & DuBois D. F. Geometric aspects of HF driven Langmuir turbulence in the ionosphere, *Nonlin. Proc. Geophys.* **10**, 151–177 (2003). https://doi.org/10.5194/npg-10-151-2003

[7] Senior, A., Rietveld, M. T., Honary, F., Singer W. & Kosch, M. J. Measurements and modelling of cosmic noise absorption changes due to radio heating of the D-Region ionosphere, *J. Geophys. Res.* **116**, A04310 (2011). https://doi.org/10.1029/2010JA016189

[8] Fried, B. D. & Conte, S. D. The Plasma Dispersion Function: The Hilbert Transform of the Gaussian (Academic Press, New York, 1961).

[9] Bernhardt, P. A. *et al.* Determination of the electron temperature in the modified ionosphere over HAARP using the HF pumped Stimulated Brillouin Scatter (SBS) emission lines, *Ann. Geophys*. **27**, 4409–4427 (2009). https://doi.org/10.5194/angeo-27-4409-2009

[10] Bernhardt, P. A. *et al.* Stimulated Brillouin scatter in a magnetized ionospheric plasma, *Phys. Rev. Lett.* **104**, 165004 (2010). https://doi.org/10.1103/PhysRevLett.104.165004

[11] Mahmoudian, A., Nossa, E., Isham, B., Bernhardt, P. A., Briczinski, S. J., & Sulzer, M. NSEE yielding electron temperature measurements at the Arecibo Observatory. *J. Geophys. Res. Space Physics*, **124**, 3699–3708 (2019). https://doi.org/10.1029/2019JA026594

[12] Bernhardt, P. A., Selcher, C. A. & Kowtha, S. Electron and ion Bernstein waves excited in the ionosphere by high power EM waves at the second harmonic of the electron cyclotron frequency, *Geophys. Res. Lett.* **38**, L19107 (2011). https://doi.org/10.1029/2011GL049390

[13] Samimi, A. *et al.* On ion gyro-harmonic structuring in the stimulated electromagnetic emission spectrum during second electron gyro-harmonic heating, *Ann. Geophys.* **30**, 1584-1594 (2012). https://doi.org/10.5194/angeo-30-1587-2012

[14] Lundborg, B. & Thidé, B. Standing wave pattern of HF radio waves in the ionospheric reflection region: 2. Applications, *Radio Sci.* **21**(3), 486– 500 (1986). https://doi.org/10.1029/RS021i003p00486

[15] Bingham, R. & Lashmore-Davies, C. N. Self-modulation and filamentation of electromagnetic waves in a plasma, *Nuclear Fusion* **16**(1), 67–72 (1976). https://doi.org/10.1088/0029-5515/16/1/007

[16] Ginzburg, V. L. *The propagation of Electromagnetic Waves in Plasmas* (Pergamon Press, New York, 1964).

[17] Ratcliffe, J. A. *The Magneto-Ionic Theory and Its Applications to the Ionosphere* (Cambridge University Press, 1962) Chapter 4.

[18] Leyser, T. B. Stimulated electromagnetic emissions by high-frequency electromagnetic pumping of the ionospheric plasma, *Space Sci. Rev.* **98**(3–4), 223–328 (2001). https://doi.org/10.1023/A:1013875603938

[19] Fejer, J. A. & Kopka, H. The Effect of Plasma instabilities on the ionospherically reflected wave from a high-power transmitter, *J. Geophys. Res.* **86**, 5746–5750 (1981). https://doi.org/10.1029/JA086iA07p05746

[20] Mjølhus, E. Anomalous absorption and reflection in ionospheric radio modification experiments, *J. Geophys. Res.* **90**(A5), 4269–4279 (1985). https://doi.org/10.1029/JA090iA05p04269

[21] Mjølhus, E. Theoretical model for long time stimulated electromagnetic emission generation in ionospheric radio modification experiments, *J. Geophys. Res.* **103**(A7), 14711– 14729 (1998). https://doi.org/10.1029/98JA00927

[22] Eliasson, B. & Papadopoulos, K. Numerical study of anomalous absorption of O mode waves on magnetic field-aligned striations, *Geophys. Res. Lett.* **42**, 2603–2611 (2015). https://doi.org/10.1002/2015GL063751

[23] Grach, S. M., Sergeev, E. N., Mishin, E. V. & Shindin, A. V. Dynamic properties of ionospheric plasma turbulence driven by high-power high-frequency radiowaves, *Phys. Usp.* **59**(11), 1091–1128 (2016). https://doi.org/10.3367/UFNe.2016.07.037868

[24] Nishikawa, K. Parametric excitation of coupled waves I. General formulation, *J. Phys. Soc. Japan* **24**(4), 916-922 (1968). https://doi.org/10.1143/JPSJ.24.916

[25] Sanmartin, J. R. Electrostatic plasma instabilities excited by a high-frequency electric field, *Phys. Fluids* **13**(6), 1533-1542 (1970). https://doi.org/10.1063/1.1693114

[26] Lashmore-Davies, C. N. The coupled mode approach to nonlinear wave interactions and parametric instabilities, *Plasma Physics* **17**(4), 281–303 (1975). https://doi.org/10.1088/0032-1028/17/4/005

[27] Eliasson, B. & Papadopoulos, K. HF wave propagation and induced ionospheric turbulence in the magnetic equatorial region, *J. Geophys. Res. Space Physics* **121**, 2727-2742 (2016). https://doi.org/10.1002/2015JA022323

[28] Bingham, R. & Lashmore-Davies, C. N. On the nonlinear development of the Langmuir modulational instability, *J. Plasma Phys.* **21**(1), 51–69 (1979). https://doi.org/10.1017/S0022377800021644

[29] Stenflo, L. & Shukla, P. K. Parametric instabilities and nucleation of coupled Langmuir and acoustic waves in a weakly-ionized plasma, *Astrophys. Space Sci.* **172**, 317-320 (1990). https://doi.org/10.1007/BF00643325

[30] Fejer, J. A. Physical processes of ionospheric heating experiments, *Adv. Space Res.* **8**(1), 261-270 (1988). https://doi.org/10.1016/0273-1177(88)90371-7

[31] Robinson, P. A. Nonlinear wave collapse and strong turbulence, *Rev. Mod. Phys.* **69**(2), 507–573 (1997). https://doi.org/10.1103/RevModPhys.69.507

[32] Djuth, F. T. & D. F. DuBois, Temporal development of HF-excited Langmuir and ion turbulence at Arecibo, *Earth Moon Planets* **116**, 19–53 (2015). https://doi.org/10.1007/s11038-015-9458-x.

[33] Duncan, L. M. & Sheerin, J. P. High-resolution studies of the HF ionospheric modification interaction region, *J. Geophys. Res.* **90**(A9), 8371– 8376 (1985). https://doi.org/10.1029/JA090iA09p08371

[34] Dawson, J. & Oberman C. high-frequency conductivity and the emission and absorption coefficients of a fully ionized plasma, *Phys. Fluids* **5**, 517 (1962). https://doi.org/10.1063/1.1706652

[35] Kruer, W. L. & Dawson, J. M. Anomalous high-frequency resistivity of a plasma, *Phys. Fluids* **15**(3), 446–453 (1972). https://doi.org/10.1063/1.1693927

[36] Faehl, R. J. & Kruer, W. L. Laser light absorption by short wavelength ion turbulence, *Phys. Fluids* **20**(1), 55-60 (1977). https://doi.org/10.1063/1.861697

[37] Kruer, W. L. Laser plasma interactions with intensities from 10^{12}–10^{21} W/cm^2, *Phys. Plasmas* **10**(5), 2087—2092 (2003). https://doi.org/10.1063/1.1559005

[38] Cohen, R. & Whitehead, J. D. Radio-reflectivity detection of artificial modification of the ionospheric F layer, *J. Geophys. Res.* **75**(31), 6439–6445 (1970). https://doi.org/10.1029/JA075i031p06439

[39] Stubbe, P., Kopka, H., Jones, T. B. & Robinson, T. Wide band attenuation of radio waves caused by powerful HF waves: Saturation and dependence on ionospheric variability, *J. Geophys. Res.* **87**(A3), 1551–1555 (1982). https://doi.org/10.1029/JA087iA03p01551

[40] Getmantsev, G. G., *et al.* Some results of investigations of nonlinear phenomena in the F-layer of the ionosphere, *JETP Lett.* **18**, 364–366 (1973). http://jetpletters.ru/ps/1569/article_24047.shtml

[41] Grach, S. M., Mityakov, N. A., Rapoport, V. O. & Trakhtengertz, V. Yu. Thermal parametric turbulence in a plasma, *Physica D* **2**(1), 102–106 (1981). https://doi.org/10.1016/0167-2789(81)90063-4

[42] Gurevich, A. V., Zybin, K. B. & Lukyanov, A. V. Stationary striations developed in the ionospheric modification, *Phys. Rev. Lett.* **75**(13), 2622 (1995). https://doi.org/10.1103/PhysRevLett.75.2622

[43] Istomin, Ya. N. & Leyser, T. B. Small-scale magnetic field-aligned density irregularities excited by a powerful electromagnetic wave, *Phys. Plasmas* **4**, 817–828 (1997). https://doi.org/10.1063/1.872175

[44] Honary, F., Stocker, A. J., Robinson, T. R., Jones, T. B. & Stubbe, P. Ionospheric plasma response to HF radio waves operating at frequencies close to the third harmonic of the electron gyrofrequency, *J. Geophys. Res.* **100**(A11), 21489– 21501 (1995). https://doi.org/10.1029/95JA02098

[45] Honary, F. *et al.* First direct observations of the reduced striations at pump frequencies close to the electron gyroharmonics, *Ann. Geophys.* **17**, 1235–1238 (1999). https://doi.org/10.1007/s00585-999-1235-6

[46] Mjølhus E. On the small scale striation effect in ionospheric radio modification experiments near harmonics of the electron gyro frequency, *J. Atm. Terr. Phys.* **55**(6), 907-918 (1993). https://doi.org/10.1016/0021-9169(93)90030-3

[47] Leyser, T. B., Thidé, B., Waldenvik, M., Veszelei, E., Frolov, V. L., Grach, S. M. & Komrakov, G. P. Downshifted maximum features in stimulated electromagnetic emission spectra, *J. Geophys. Res.* **99**(A10), 19555–19568 (1994). https://dx.doi.org/10.1029/94JA01399

[48] Grach, S.M., Thidé, B. & Leyser, T.B. Plasma waves near the double resonance layer in the ionosphere, *Radiophys. Quantum Electron.* **37**, 392–402 (1994). https://doi.org/10.1007/BF01045689

[49] Frolov, V. L. *et al*., Ponderomotive narrow continuum (NCp) component in stimulated electromagnetic emission spectra, *J. Geophys. Res.* **109**, A07304 (2004). https://doi.org/10.1029/2001JA005063

[50] Thidé, B., Sergeev, E. N., Grach, S. M., Leyser, T. B. & Carozzi, T. D. Competition between Langmuir and upper-hybrid turbulence in a high-frequency-pumped ionosphere, *Phys. Rev. Lett.* **95**, 255002 (2005). https://doi.org/10.1103/PhysRevLett.95.255002

[51] Carozzi, T. D., *et al.* Stimulated electromagnetic emissions during pump frequency sweep through fourth electron cyclotron harmonic, *J. Geophys. Res.* **107**(A9), 1253 (2002). https://doi.org/10.1029/2001JA005082

[52] Gurevich, A. Nonlinear effects in the ionosphere. *Phys. Usp.* **50**, 1091–1121 (2007). https://doi.org/10.1070/PU2007v050n11ABEH006212

[53] Leyser, T. B. & Wong, A. Y. Powerful electromagnetic waves for active environmental research in geospace. *Rev. Geophys*. **47**(1) (2009). https://doi.org/10.1029/2007rg000235

[54] Streltsov, A. V., *et al.* Past, present and future of active radio frequency experiments in space, *Space Sci. Rev.* **218**, 118 (2018). https://doi.org/10.1007/s11214-018-0549-7

[55] Derblom, H. *et al.* Tromsø Heating Experiments: Stimulated emission at HF pump harmonic and subharmonic frequencies, *J. Geophys. Res.*, **94**(A8), 10111– 10120 (1989). https://doi.org/10.1029/JA094iA08p10111

[56] Thidé, B. Stimulated scattering of large amplitude waves in the ionosphere: experimental results, *Phys. Scr.* **1990**(T30), 170–180 (1990). https://doi.org/10.1088/0031-8949/1990/T30/023
